# Supplementary material for: An open-source framework for large-scale, flexible evaluation of biomedical text mining systems
Source: J Biomed Discov Collab. 2008 Jan 29;3:1. doi: 10.1186/1747-5333-3-1 (PMC2276192; doi:10.1186/1747-5333-3-1)
Supplement: Additional file 1 — Evaluation platform source code. The additional file contains the software evaluation framework discussed in this paper. The most current release can be downloaded from SourceForge.net [1]. See the accompanying README file for instructions on installation and use. Also included in the distribution is a collection of UIMA wrappers for some commonly used BioNLP tools and annotated corpora. [file 1747-5333-3-1-S1.zip › bionlp_uima_repository-1.0.1/docs/api/edu/uchsc/ccp/uima/ae/nlp/opendmap/OpenDmapAnnotator.html]

OpenDmapAnnotator (Center for Computational Pharmacology Util/UIMA API)


|  |  |  |
| --- | --- | --- |
| **Overview** \| **Package** \| **Class** \| **Tree** \| **Deprecated** \| **Index** \| **Help** | |  |
| **PREV CLASS**   NEXT CLASS | **FRAMES**    **NO FRAMES**     **All Classes** |  |
| SUMMARY: NESTED \| FIELD \| CONSTR \| METHOD | DETAIL: FIELD \| CONSTR \| METHOD |  |


---


## edu.uchsc.ccp.uima.ae.nlp.opendmap Class OpenDmapAnnotator

```
java.lang.Object
  org.apache.uima.analysis_engine.annotator.Annotator_ImplBase
      org.apache.uima.analysis_engine.annotator.JTextAnnotator_ImplBase
          edu.uchsc.ccp.uima.ae.nlp.opendmap.OpenDmapAnnotator
```

**All Implemented Interfaces:**: org.apache.uima.analysis\_engine.annotator.BaseAnnotator, org.apache.uima.analysis\_engine.annotator.JTextAnnotator

**Direct Known Subclasses:**: CCPOpenDMAP\_AE

---

``` public abstract class OpenDmapAnnotator extends org.apache.uima.analysis_engine.annotator.JTextAnnotator_ImplBase ```

This class is a wrapper for OpenDMAP to help fit it into the
UIMA framework.

Right now this class hides many of the details involved in
setting up the parser, connecting it to Protege, and loading
pattern definitions.

To build a UIMA annotator with OpenDMAP, extend this class.

**Author:**
:   R. James Firby

---

**Constructor Summary**

`OpenDmapAnnotator()`
  
          Create a new, empty OpenDMAP Annotator.


| **Method Summary** | |
| --- | --- |
| `void` | `addPatternFilename(String filename)`             Add the name of an OpenDMAP pattern file to be loaded into the parser when this annotator is initialized. |
| `void` | `initialize(org.apache.uima.analysis_engine.annotator.AnnotatorContext context)`             Initialize the OpenDMAP parser and load the Protege project and the pattern files. |
| `void` | `process(org.apache.uima.jcas.JCas jcas, org.apache.uima.analysis_engine.ResultSpecification resultSpec)`             When the UIMA pipeline signals it is time to run this annotator, call user code to get tokens from the jcas, call the parser, and then write annotations back to the jcas. |
| `abstract  void` | `process(edu.uchsc.ccp.opendmap.Parser parser, org.apache.uima.jcas.JCas jcas, org.apache.uima.analysis_engine.ResultSpecification resultSpec)`             Process the JCas as part of a UIMA pipeline. |
| `void` | `setProtegeProjectFilename(String filename)`             Set the Protege Project file to be loaded into the OpenDMAP parser when this annotator is initialized. |

**Methods inherited from class org.apache.uima.analysis\_engine.annotator.Annotator\_ImplBase**

`destroy, finalize, getContext, getTypeSystem, reconfigure, typeSystemInit`

**Methods inherited from class java.lang.Object**

`clone, equals, getClass, hashCode, notify, notifyAll, toString, wait, wait, wait`

**Methods inherited from interface org.apache.uima.analysis\_engine.annotator.BaseAnnotator**

`destroy, reconfigure, typeSystemInit`

**Constructor Detail**

### OpenDmapAnnotator

```
public OpenDmapAnnotator()
```

:   Create a new, empty OpenDMAP Annotator.


**Method Detail**

### setProtegeProjectFilename

```
public void setProtegeProjectFilename(String filename)
```

:   Set the Protege Project file to be loaded into the
    OpenDMAP parser when this annotator is initialized.
    Only one project file may be used for each annotator.

    :   **Parameters:**: `filename` - The name of the Protege project file

---


### addPatternFilename

```
public void addPatternFilename(String filename)
```

:   Add the name of an OpenDMAP pattern file to be loaded
    into the parser when this annotator is initialized.
    Any number of pattern files may be added.

    :   **Parameters:**: `filename` - The name of one pattern file

---


### initialize

```
public void initialize(org.apache.uima.analysis_engine.annotator.AnnotatorContext context)
                throws org.apache.uima.analysis_engine.annotator.AnnotatorInitializationException
```

:   Initialize the OpenDMAP parser and load the Protege
    project and the pattern files.

    :   **Specified by:**: `initialize` in interface `org.apache.uima.analysis_engine.annotator.BaseAnnotator` **Overrides:**: `initialize` in class `org.apache.uima.analysis_engine.annotator.Annotator_ImplBase`
    :   **Throws:**: `org.apache.uima.analysis_engine.annotator.AnnotatorInitializationException`

---


### process

```
public void process(org.apache.uima.jcas.JCas jcas,
                    org.apache.uima.analysis_engine.ResultSpecification resultSpec)
             throws org.apache.uima.analysis_engine.annotator.AnnotatorProcessException
```

:   When the UIMA pipeline signals it is time to run this
    annotator, call user code to get tokens from the jcas,
    call the parser, and then write annotations back to the
    jcas.

    :   **Throws:**: `org.apache.uima.analysis_engine.annotator.AnnotatorProcessException`

---


### process

```
public abstract void process(edu.uchsc.ccp.opendmap.Parser parser,
                             org.apache.uima.jcas.JCas jcas,
                             org.apache.uima.analysis_engine.ResultSpecification resultSpec)
```

:   Process the JCas as part of a UIMA pipeline. Ie. get input
    tokens for OpenDMAP from the JCas, run the OpenDMAP parser,
    process the results and write appropriate annotations back
    to the JCas.

    :   **Parameters:**: `parser` - The OpenDMAP parser to use for parsing.: `jcas` - The JCas holding input tokens and output annotations.: `resultSpec` - The type of result expected to be put into the JCas.


---


|  |  |  |
| --- | --- | --- |
| **Overview** \| **Package** \| **Class** \| **Tree** \| **Deprecated** \| **Index** \| **Help** | |  |
| **PREV CLASS**   NEXT CLASS | **FRAMES**    **NO FRAMES**     **All Classes** |  |
| SUMMARY: NESTED \| FIELD \| CONSTR \| METHOD | DETAIL: FIELD \| CONSTR \| METHOD |  |


---
